# Pathogens attributed to central-line–associated bloodstream infections in US acute-care hospitals during the first year of the coronavirus disease 2019 (COVID-19) pandemic

**DOI:** 10.1017/ice.2022.16

**Published:** 2022-02-08

**Authors:** Lindsey M. Weiner-Lastinger, Kathryn Haass, Cindy Gross, Denise Leaptrot, Emily Wong, Hsiu Wu, Margaret A. Dudeck

**Affiliations:** 1 Centers for Disease Control and Prevention, Atlanta, Georgia; 2 CACI, Atlanta, Georgia; 3 Leidos, Atlanta, Georgia

## Abstract

To assess potential changes in the pathogens attributed to central-line–associated bloodstream infections between 2019 and 2020, hospital data from the National Healthcare Safety Network were analyzed. Compared to 2019, increases in the proportions of pathogens identified as *Enterococcus faecalis* and coagulase-negative staphylococci were observed during 2020.

During 2020, hospitals saw unprecedented increases in critically ill patients as coronavirus disease 2019 (COVID-19) spread across the United States. In response, hospitals were often required to modify their operations, services provided, and patient care practices.^1^ Several studies have documented an alarming increase in device utilization and healthcare-associated infections (HAIs) in the United States during 2020, particularly in intensive care units (ICUs)^2–4^; however, studies evaluating changes in HAI pathogens during the pandemic have been limited to a small number of facilities. To assess changes in the common pathogens reported from central-line–associated bloodstream infections (CLABSIs) at the national level between 2019 and 2020, we examined data reported to the National Healthcare Safety Network (NHSN) by acute-care hospitals.

## Methods

For each CLABSI, hospitals are required to report 1–3 pathogens and select antimicrobial susceptibility results to NHSN.^5^ CLABSI pathogens identified in adult ICUs and wards in 2019 and 2020 were analyzed. “Wards” included all adult non–critical-care units such as step-down and mixed-acuity units, excluding inpatient rehabilitation units.

The 15 pathogens most frequently associated with CLABSIs in 2019 and 2020 were identified, and their frequencies and ranks within each location type were calculated. Pathogen distributions were also reviewed among the subset of hospitals that performed continuous HAI surveillance in both 2019 and 2020, with no difference in results observed. Although COVID-19 patient status was an optional field for data entry in NHSN, the distribution of pathogens among COVID-19 ICU patients was assessed. Vancomycin resistance among *Enterococcus* (VRE) and methicillin resistance among *Staphylococcus aureus* (MRSA) were measured by calculating the percentage of tested pathogens that were resistant. A mid-*P* exact test result ≤.05 was used to identify significant differences.

## Results

Most CLABSIs in 2019 (89.4%) and 2020 (89.1%) had a single pathogen identified, with no substantial change in the proportion of CLABSIs that were polymicrobial.

### ICUs

In total, 7,675 ICU CLABSI pathogens were reported from 1,560 hospitals during 2019. The most common pathogens were *Candida* (29.3%), coagulase-negative staphylococci (CNS) (13.3%), and *Enterococcus faecium* (8.1%) (Table 1). In 2020, 12,635 pathogens were reported by 1,906 hospitals, and *Candida* (27.8%), CNS (18.2%), and *Enterococcus faecalis* (15.0%) were the 3 most frequently reported species.

**Table 1.** Frequency and Distribution of the 15 CLABSI Pathogens Most Frequently Reported to the NHSN from Adult ICUs in 2019 and 2020

Pathogen	2019			2020		
	No.	%	Rank	No.	%	Rank
All *Candida* spp	2,246	29.3	1	3,513	27.8	1
Coagulase-negative staphylococci	1,023	13.3	2	2,296	18.2	2
*Enterococcus faecium*	622	8.1	3	787	6.2	5
*Staphylococcus aureus*	613	8.0	4	882	7.0	4
*Enterococcus faecalis*	590	7.7	5	1,895	15.0	3
Selected *Klebsiella* spp^a,b^	432	5.6	6	541	4.3	6
*Escherichia coli*	306	4.0	7	333	2.6	8
*Enterobacter* spp^b^	263	3.4	8	228	1.8	9
*Pseudomonas aeruginosa*	235	3.1	9	340	2.7	7
*Serratia* spp	179	2.3	10	196	1.6	11
Other *Enterococcus* spp^c^	135	1.8	11	205	1.6	10
*Acinetobacter* spp	101	1.3	12	120	0.9	13
*Bacteroides* spp	85	1.1	13	102	0.8	14
Yeast, not specified	81	1.1	14	154	1.2	12
*Proteus* spp	74	1.0	15	89	0.7	16
Viridans group streptococci	55	0.7	16	93	0.7	15
Other pathogen	635	8.3		861	6.8	
**Total**	7,675	100.0		12,635	100.0	

Note. CLABSI, central-line–associated bloodstream infection; NHSN, National Healthcare Safety Network; ICU, intensive care unit.

a Includes *K. oxytoca* and *K. pneumoniae*. For 2020, this group also includes *K. aerogenes.*

b *K. aerogenes* (formerly known as *Enterobacter aerogenes*) is classed in the *Enterobacter* spp group in 2019 and the *Klebsiella* spp group in 2020.

c The group ‘other *Enterococcus* spp’ combines enterococci identified to the species level, excluding *E. faecium* and *E. faecalis*, and enterococci for which the species was not reported.

A large increase in the proportion of ICU CLABSI pathogens identified as CNS and *E. faecalis* were noted in 2020 compared to 2019. The increase in absolute number of *E. faecalis* CLABSIs was widespread; 388 hospitals reported at least 1 *E. faecalis* ICU CLABSI pathogen in 2019, compared to 848 hospitals in 2020 (data not shown). The reporting of *E. faecalis* varied by month in 2020, with the proportion of pathogens identified as *E. faecalis* ranging from 8%–9% (January–March) to 17%–18% (November and December). Little variation by month was observed in 2019, when the proportion of *E. faecalis* pathogens ranged from 6% to 9% for almost all months in the year.

In 2019, 5.9% of tested *E. faecalis* were resistant to vancomycin (VRE); the resistance percentage was significantly lower in 2020, at 3.0% (Table A1).

### Wards

In total, 1,821 hospitals reported 14,508 CLABSI pathogens from wards in 2019, of which *Candida* (12.1%), *S. aureus* (11.8%), and *Escherichia coli* (11.5%) were the most frequently reported (Table 2). In 2020, 1,848 hospitals reported 13,943 pathogens, and CNS replaced *E. coli* to become the third most common pathogen (11.1%). Increases were observed in the proportion of pathogens in 2020 that were CNS and *E. faecalis* compared to 2019. The percentage of *E. faecalis* that were resistant to vancomycin was significantly lower in 2020 than 2019 (5.2% vs 7.6%) (Table A1).

**Table 2.** Frequency and Distribution of the 15 CLABSI Pathogens Most Frequently Reported Reported to NHSN from Adult Wards^a^ in 2019 and 2020

Pathogen	2019			2020		
	No.	%	Rank	No.	%	Rank
All *Candida* spp.	1,758	12.1	1	1,822	13.1	1
*Staphylococcus aureus*	1,710	11.8	2	1,681	12.1	2
*Escherichia coli*	1,666	11.5	3	1,502	10.8	4
Coagulase-negative staphylococci	1,453	10.0	4	1,544	11.1	3
Selected *Klebsiella* spp^b,c^	1,300	9.0	5	1,187	8.5	5
*Enterococcus faecalis*	957	6.6	6	1,081	7.8	6
*Enterococcus faecium*	880	6.1	7	809	5.8	7
Viridans group streptococci	707	4.9	8	647	4.6	8
*Pseudomonas aeruginosa*	691	4.8	9	573	4.1	9
*Enterobacter* spp^c^	566	3.9	10	416	3.0	10
*Serratia* spp.	225	1.6	11	199	1.4	13
Other *Enterococcus* spp^d^	213	1.5	12	218	1.6	11
*Bacteroides* spp	166	1.1	13	216	1.5	12
*Stenotrophomonas maltophilia*	146	1.0	14	127	0.9	15
*Proteus* spp	137	0.9	15	130	0.9	14
Other pathogen	1,933	13.3		1,791	12.8	
**Total**	14,508	100.0		13,943	100	

Note. CLABSI, central-line–associated bloodstream infection; NHSN, National Healthcare Safety Network.

a Includes all non–critical-care unit types, including specialty care areas, step-down units, and mixed-acuity units.

b Includes *K. oxytoca* and *K. pneumoniae*. For 2020, this group also includes *K. aerogenes.*

c *K. aerogenes* (formerly known as *Enterobacter aerogenes*) is classed in the *Enterobacter* spp. group in 2019 and the *Klebsiella* spp. group in 2020.

d The group ‘other *Enterococcus* spp’ combines enterococci identified to the species level, excluding *E. faecium* and *E. faecalis*, and enterococci for which the species was not reported.

### CLABSIs in COVID-19 ICU patients

Data on COVID-19 patient status were available for 4,232 (33.5%) of ICU CLABSI pathogens, with 2,787 (65.9% of those with data) occurring in patients with confirmed or suspected COVID-19 (Table A2). *Candida* (28.9%), *E. faecalis* (21.1%), and CNS (19.7%) were the 3 most frequently reported CLABSI pathogens among ICU patients with COVID-19.

## Discussion

This paper describes the CLABSI pathogens commonly isolated during the first year of the COVID-19 pandemic, using data from almost all US hospitals.^6^ Our results showed that the common pathogens among COVID-19 ICU patients at a national level, particularly *E. faecalis* and CNS, were consistent with results from local studies.^7–10^

Even though the stark increase in *E. faecalis* pathogens reported in 2020 was unexpected, an increase in *Enterococcus* BSIs in 2020, compared to 2018–2019, was also observed in a hospital in northern Italy.^7^ These results, along with substantially higher proportions of *E. faecalis* identified in November and December 2020, during which a large number of COVID-19 hospitalizations occurred in the United States,^11^ suggest that COVID-19 patients and/or patients hospitalized during times of heightened COVID-19 burden may be particularly susceptible to CLABSIs caused by *E. faecalis*. The reasons for this are unclear, but several local studies from the United States and Italy identified *Enterococcus* as a common BSI pathogen among COVID-19 patients.^7–10^

In addition to host factors, changes in the amount and overall pattern of antibiotic use for hospitalized patients in 2020 could have contributed to a rise in *E. faecalis.* Giacobbe et al^8^ reported that almost all COVID-19 patients in their 1,200-bed hospital were treated with a cephalosporin, and an increase in antibiotic use, especially ceftriaxone, was observed in 2 large US hospital cohorts during 2020.^12^ The additional antibiotic use in 2020, or other antibiotic effects, may have contributed to changes in the selection pressure for pathogens in hospitals that favored the growth of *E. faecalis*. Interestingly, increases in *E. faecium* were not observed during 2020. Additional research is needed to understand the mechanism behind the increases in *E. faecalis* and to more fully explore the impact of the COVID-19 pandemic on pathogens and antimicrobial resistance patterns in hospitals.

CNS was the second most reported pathogen for ICU CLABSIs in 2019 and 2020, with a marked increase in 2020. Due to the surge of case load and relative scarce healthcare resources early in the pandemic, inadequate adherence to aseptic blood culture collection technique may have resulted in some increases in CNS isolates.^13–15^ However, the NHSN CLABSI definition includes stipulations to reduce the impact of contamination by offering separate criteria for common commensals and known pathogens; thus, the increase in CNS CLABSIs during 2020 is unlikely to have been caused by contamination alone.^5^

This study had several limitations. All data from adult locations were analyzed, including data from pediatric patients housed in adult locations at the time of their infection. The CMS granted a reporting exception for the first half of 2020, leading some hospitals to pause HAI reporting to the NHSN. Any underestimation in the number of pathogens during 2020 is assumed to be minimal due to the high volume of reporting that continued throughout the year.^4^ It was optional for hospitals to report patient COVID-19 status to the NHSN. Given the limited responses available, we acknowledge that the pathogen distribution among COVID-19 ICU patients is not representative of all COVID-19 ICU patients who experienced an HAI.

Compared to the pre–COVID-19 period, we identified national increases during 2020 in the proportion of CLABSIs caused by *E. faecalis* and CNS. Infection prevention professionals are encouraged to review the common pathogens and antimicrobial resistance patterns in their hospitals and jurisdictions to identify opportunities to strengthen HAI prevention and antimicrobial stewardship efforts.

## Supplementary material

For supplementary material accompanying this paper visit https://doi.org/10.1017/ice.2022.16. click here to view supplementary material

## APPENDIX

**Table A1.** The Percentage of CLABSI Pathogens Resistant (%R) to Vancomycin (VRE) or Methicillin (MRSA) in 2019 and 2020, by Location

Location, Pathogen	2019			2020			
	No. Tested	% Tested	% R^a^	No. Tested	% Tested	%R^a^	*P* Value
Adult ICUs							
* Enterococcus faecalis*	525	90.0	5.9	1,681	88.7	3.0	.004
* E. faecium*	574	92.3	81.5	713	90.6	77.7	.091
* Staphylococcus aureus*	531	86.6	47.8	758	85.9	47.6	.941
Adult Wards^b^							
* E. faecalis*	854	89.2	7.6	939	86.9	5.2	.039
* E. faecium*	811	92.2	72.5	713	88.1	70.3	.336
* S. aureus*	1,498	87.6	46.2	1,432	85.2	48.3	.249

Note. ICU, intensive care unit; CLABSI, central-line–associated bloodstream infection; VRE, vancomycin-resistant *Enterococcus*; MRSA, methicillin-resistant *Staphylococcus aureus*.

a Percent resistance (%R) is measured for VRE or MRSA, as appropriate. VRE is defined as *Enterococcus* resistant to vancomycin. MRSA is defined as *S. aureus* resistant to methicillin, oxacillin, or cefoxitin.

b Includes all non–critical-care unit types, including specialty care areas, step-down units, and mixed-acuity units.

**Table A2.** Frequency and Distribution of the CLABSI Pathogens Most Frequently Reported Among Adult ICU Patients With Confirmed or Suspected COVID-19

Pathogen	No.	%	Rank
All *Candida* spp.	806	28.9	1
*Enterococcus faecalis*	589	21.1	2
Coagulase-negative staphylococci	550	19.7	3
*Staphylococcus aureus*	186	6.7	4
*Enterococcus faecium*	148	5.3	5
Selected *Klebsiella* spp^a^	77	2.8	6
*Pseudomonas aeruginosa*	60	2.2	7
*Escherichia coli*	48	1.7	8
Yeast, not specified	42	1.5	9
Other *Enterococcus* spp^b^	37	1.3	10
*Serratia* spp	29	1.0	11
*Acinetobacter* spp	20	0.7	12
*Enterobacter* spp	19	0.7	13
Viridans group streptococci	16	0.6	14
*Stenotrophomonas maltophilia*	14	0.5	15
Other pathogen	146	5.2	
Total	2,787	100.0	

Note. ICU, intensive care unit; CLABSI, central-line–associated bloodstream infection; COVID-19, coronavirus disease 2019.

a Includes *K. oxytoca, K. pneumoniae,* and *K. aerogenes.*

b The group ‘other *Enterococcus* spp’ combines enterococci identified to the species level, excluding *E. faecium* and *E. faecalis*, and enterococci for which the species was not reported.
