# Supplementary material for: Pathogens attributed to central-line–associated bloodstream infections in US acute-care hospitals during the first year of the coronavirus disease 2019 (COVID-19) pandemic
Source: Infect Control Hosp Epidemiol. 2022 Feb 8:1–4. doi: 10.1017/ice.2022.16 (PMC9551181; doi:10.1017/ice.2022.16)
Supplement: Supplementary file 1 [file S0899823X22000162sup001.docx]

**
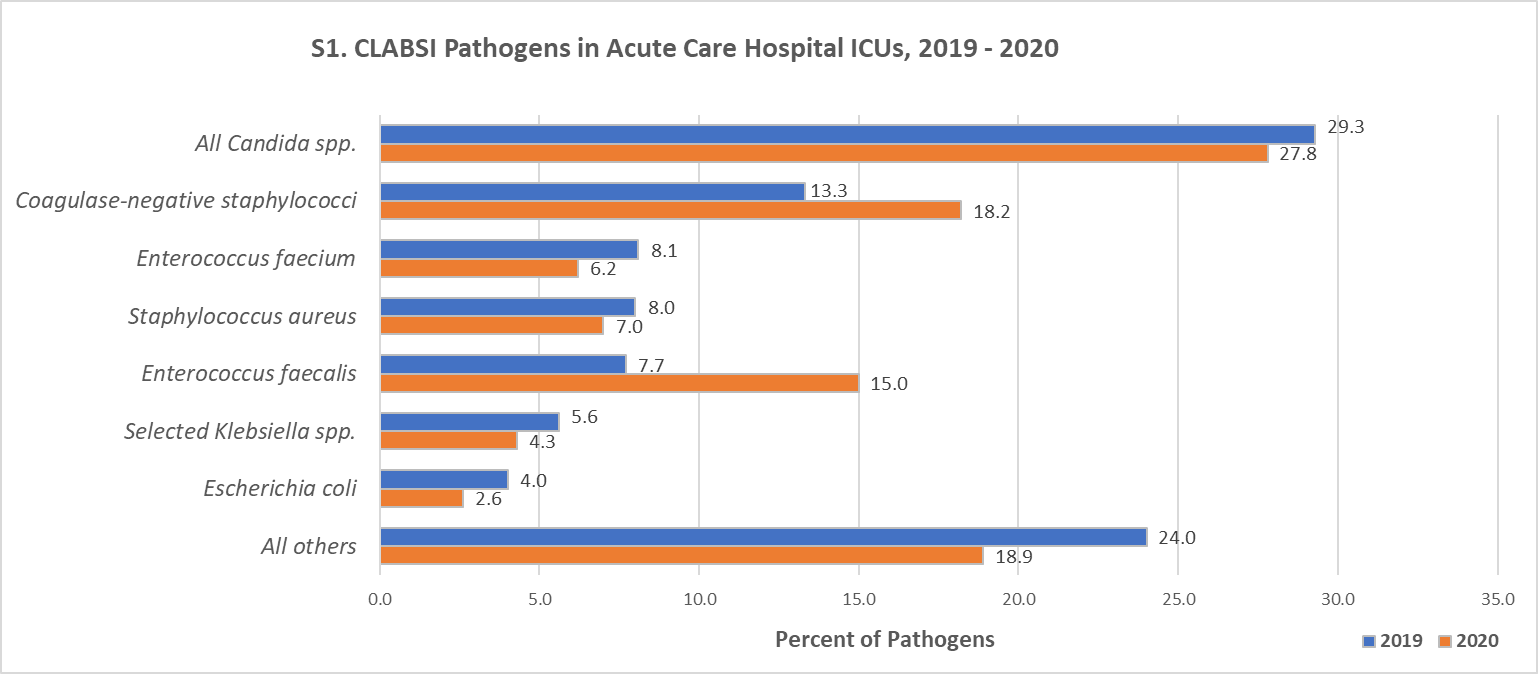
Supplement:** Pathogens attributed to central-line associated bloodstream infections in US acute care hospitals during the first year of the COVID-19 pandemic

Note. CLABSI – central line-associated bloodstream infection; ICUs – intensive care units; spp – species;


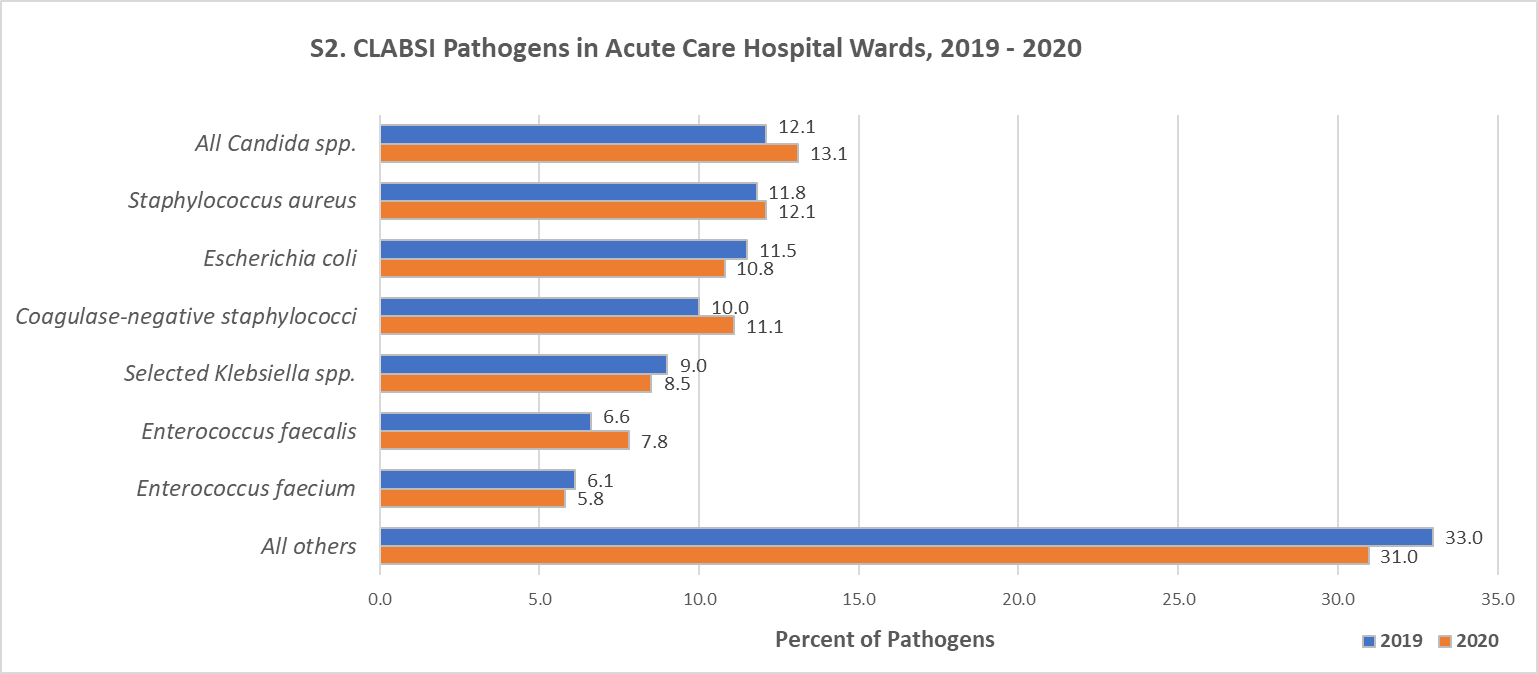


Note. CLABSI – central line-associated bloodstream infection; spp – species;
